# Efficacy and safety of trastuzumab deruxtecan in gastrointestinal malignancies: a systemic review and meta-analysis

**DOI:** 10.3389/fgstr.2025.1559934

**Published:** 2025-04-29

**Authors:** Ali Hussain, Qamar Iqbal, Sangeetha Isaac, Faisal Shariff, Ezza Tariq, Hassan Awais, Nayan Mainkar, Heidi Lynn Reis, Aakriti Arora, Akshay Deotare, Azka Tasleem, Srijan Valasapalli, Munizay Paracha, Maya Hashmi, Hamdi Battah, Mahvish Muzaffar

**Affiliations:** ^1^ Division of Hematology and Oncology, Brody School of Medicine at East Carolina University, Greenville, NC, United States; ^2^ Department of Internal Medicine, University of Toledo, Toledo, OH, United States

**Keywords:** trastuzumab deruxtecan (T-DXd), antibody-drug conjugate (ADC), gastrointestinal malignancies, safety, efficacy, HER2-positive

## Abstract

**Background:**

Trastuzumab deruxtecan (T-DXd) is an antibody-drug conjugate (ADC) that is effective in treating gastrointestinal (GI) cancers. However, the significant variability in its reported efficacy and safety profiles is likely due to differences in trial designs, patient populations, and clinical settings. This systematic review and meta-analysis aimed to consolidate current evidence on the efficacy and safety of T-DXd in human epidermal growth factor receptor 2 (HER2)-positive GI malignancies.

**Methods:**

We conducted a systematic review and meta-analysis following the Preferred Reporting Items for Systemic Reviews and Meta-Analysis guidelines (PRISMA), utilizing the Medline, Embase, Cochrane Central, and ClinicalTrials.gov databases. Out of 5,594 articles reviewed, 10 studies were ultimately included after both primary and secondary screenings, providing data on the outcomes and safety of T-DXd in HER2-positive GI malignancies. The National Institute of Health quality assessment tool was employed to evaluate the quality of the studies. Pooled analyses were performed using the ‘meta’ package (Schwarzer et al., R programming language), and proportions with 95% confidence intervals (CIs) were calculated.

**Results:**

We identified 653 patients treated with T-DXd for HER2-positive GI malignancies in 10 studies. The median age of the patients was 64.5 years (27–85) and 53% were male. The median follow-up duration was 5.9 months (0.5–30.5). The median overall survival and progression-free survival were 11.15 (1.4–20.8) and 5.6 months (2.6–8.7), respectively. The pooled objective response rate (ORR) was 36.9% (95% CI:31.5%–42.5%, I² = 41%, n = 589), with partial response and complete response rates of 35.2% (95% CI:31.1%–39.5%, I² = 0%, n = 516) and 1.3% (95% CI: 0.0%–4.7%, I² = 73%, n = 516), respectively. The median duration of response (DoR) was 7 months (0.7–22.3). Reported adverse events included anemia, febrile neutropenia, thrombocytopenia, diarrhea, nausea, interstitial lung disease/pneumonitis, heart failure, and hepatitis. For the 5.4 mg/kg dose, grade 3/4 adverse events were reported in 67 patients. For the 6.4 mg/kg dose, 146 grade 3/4 adverse events were reported.

**Conclusions:**

This meta-analysis supports the efficacy of T-DXd in patients with HER2-positive GI malignancies with a moderate ORR, even in patients who have experienced disease progression after multiple lines of therapy. Overall, T-DXd is well-tolerated, with limited severe adverse events. These findings validate existing research and underscore the need for further clinical trials, particularly in earlier lines of treatment.

## Highlights

- Antibody-drug conjugates are highly targeted drug delivery systems and T-DXd is one such anti-HER2-directed ADC with a high drug antibody ratio.
- T-DXd has demonstrated effectiveness across various HER2-positive GI cancers.
- T-DXd has a favorable safety profile in various clinical trials.
- ILD is a rare but serious side effect associated with T-DXd.
- This meta-analysis includes data from available clinical trials in HER2-positive GI cancers.

## Introduction

Human epidermal growth factor receptor 2 (HER2) is a pivotal protein implicated in the development and progression of various cancers, including gastrointestinal (GI) malignancies. Overexpression of HER2 in GI malignancies is variable with 10%–30% overexpression reported in gastric and gastroesophageal cancers and 3%–5% in colon cancer. The enriched HER2 group varies based on site and subtype with gastro-esophageal junction (GE) junction and intestinal subtypes having a higher percentage of overexpression. HER2 overexpression is more common in left-side and RAS wild-type colon cancer (1). HER2 overexpression has prognostic and predictive significance (2, 3). HER2-targeted therapies such as trastuzumab have revolutionized treatment for HER2-positive cancers, especially breast cancer. The ToGA trial was the first randomized phase III clinical trial that demonstrated the efficacy of trastuzumab with cisplatin and 5FU in advanced/metastatic gastroesophageal cancers. The other HER2-targeted monoclonal antibodies and tyrosine kinase inhibitors have been less effective in HER2-positive GI malignancies compared to breast cancer.

Acquired resistance to trastuzumab often occurs due to activating mutations in the PI3K pathway and reduced HER2 expression, further complicating treatment (3–5). HER2 expression is heterogeneous with variable expression patterns in GI cancers compared to breast cancer, with incomplete membranous staining more common in gastric cancer. The scoring system for HER2 is not standardized for GI malignancies and differs from breast cancer. Antibody-drug conjugates (ADCs) targeting HER2 offer an effective treatment option for this heterogeneous subtype of GI malignancies.

**Figure 1:**
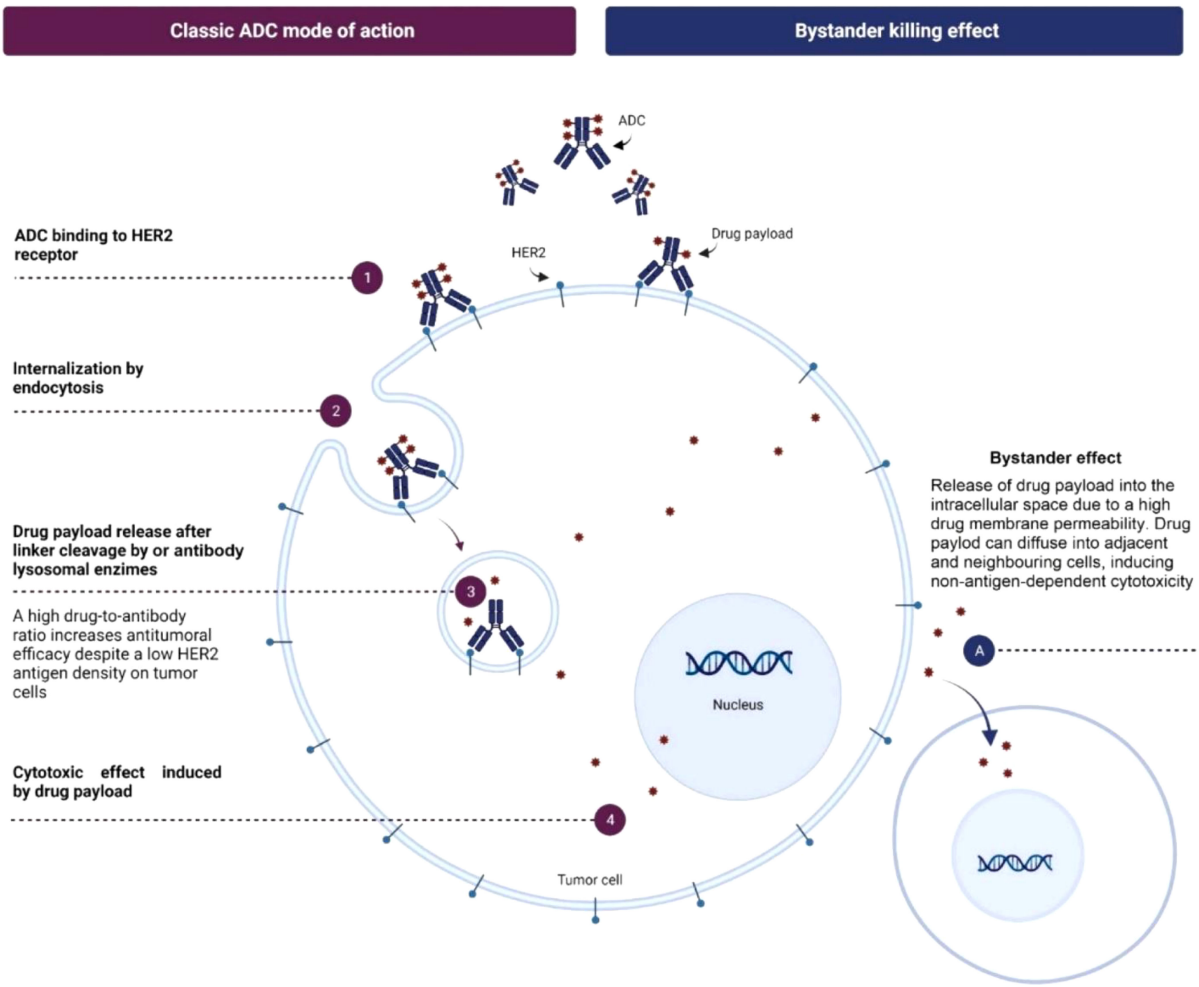
Mechanism of action of T-DXd. Adapted from "Trastuzumab deruxtecan in breast cancer" by Author M Mart�n, 2024, Elsevier. © 2024 The Authors. Published by Elsevier B.V. (Reprinted with permission).

Trastuzumab deruxtecan (T-DXd) is a novel ADC that combines a HER2-targeting monoclonal antibody with a potent topoisomerase I inhibitor linked by a stable connector. This design allows T-DXd to deliver its cytotoxic payload directly to HER2-expressing cancer cells, reducing systemic toxicity (6). T-DXd’s high drug-to-antibody ratio and bystander-killing effect enable it to target tumors with varying HER2 expression levels, making it less dependent on high HER2 expression (7). T-DXd’s topoisomerase I inhibitor payload is effective against resistance mechanisms by inducing DNA damage and cell cycle arrest independent of HER2 signaling. Unlike other ADCs, such as trastuzumab emtansine (T-DM1), T-DXd’s payload is less susceptible to efflux by ATP-binding cassette (ABC) transporters, maintaining its efficacy in resistant cells (8–10) (**Figure 1**).

Recent clinical trials have highlighted the impressive efficacy of T-DXd in treating HER2-positive GI malignancies. The DESTINY-Gastric 01 trial established T-DXd’s superiority over standard chemotherapy in patients with metastatic gastric cancer, demonstrating significant improvements in both progression-free survival (PFS) and overall survival (OS) (4). Additionally, the DESTINY-Pan Tumor 02 study supports the applicability of T-DXd across a broader range of HER2-expressing solid tumors, underscoring its potential as a transformative treatment option (11).

Despite its promising efficacy, the safety profile of T-DXd necessitates careful evaluation. Although generally well-tolerated, some patients experience severe adverse events (SAEs), particularly interstitial lung disease (ILD), and myelosuppression with grade 3 or greater adverse events (AEs) occurred in 48.3% of patients (12). Early recognition and timely management of these side effects is essential to optimize patient outcomes.

This systematic review aims to assess the safety and efficacy data of T-DXd in treating GI malignancies from currently available studies.

## Methods

### Search criteria

This systematic review and meta-analysis were conducted following the methodology outlined in the Cochrane Handbook of Systematic Reviews of Interventions (13) and was conducted according to the Preferred Reporting Items for Systemic Reviews and Meta-Analysis (PRISMA) 2020 guidelines (14). A population, intervention, comparison, and outcome (PICO) table was developed, and Librarian H.R. developed a comprehensive literature search in consultation with the research team. Another librarian peer-reviewed the search using a modified PRESS (15). The search was run from 1/1/2015 to 2/22/2024 in Medline via PubMed, Embase via Embase.com, Cochrane Central via OVID, and ClinicalTrials.gov using keywords and controlled vocabulary terms. The time filter beginning in 2015 was applied after a discussion with the research team given the first T-DXd FDA approval was in December 2019. The entire search strategy can be found at http://hdl.handle.net/10342/13380. The search results were uploaded to the screening software Covidence (https://www.covidence.org/). Duplicates were removed using the automated duplicate checker within Covidence. The reference list of included studies was also checked for additional relevant studies.

### Selection criteria

**Figure 2:**
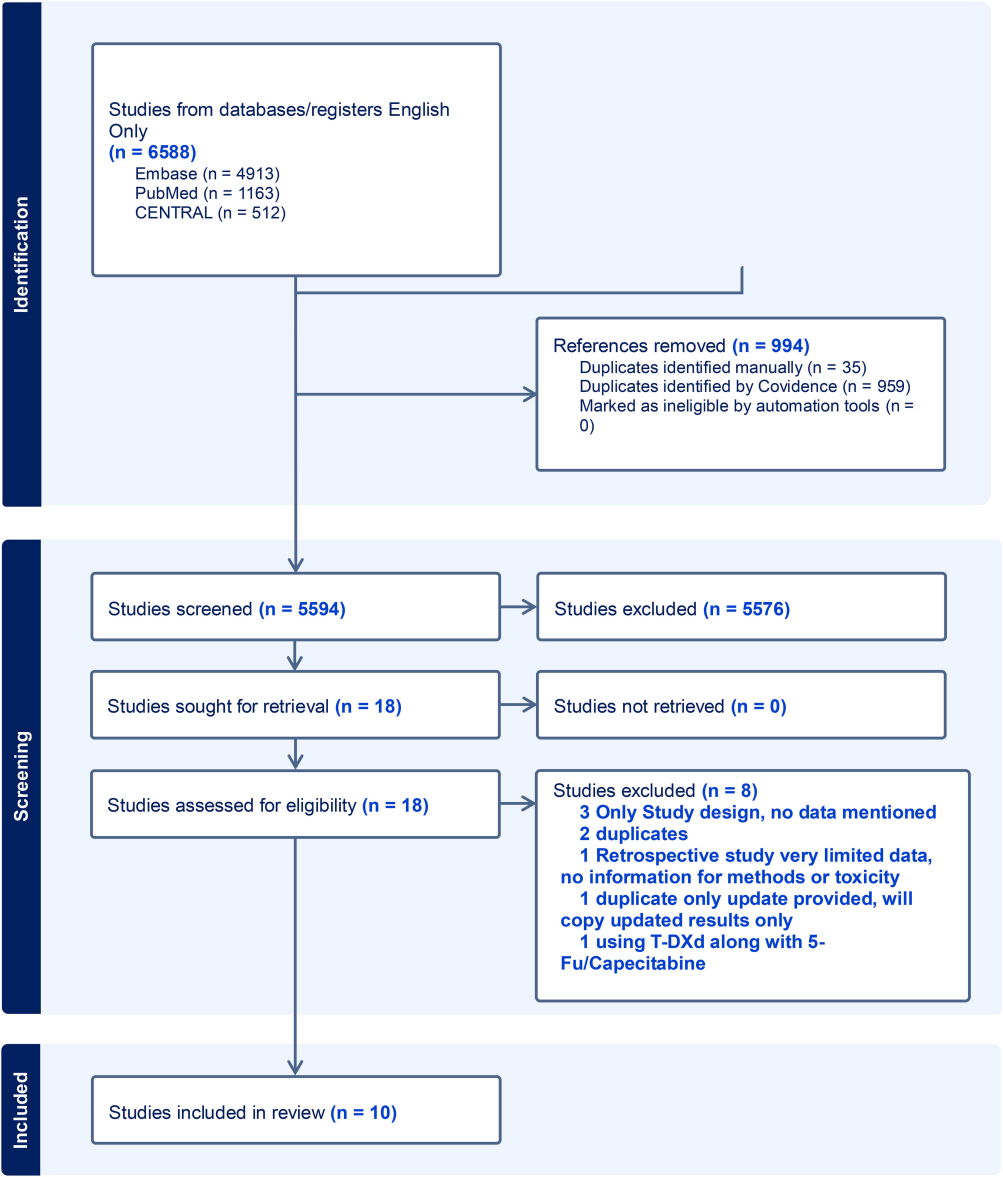
PRISMA flow chart.

After removing duplicates, 5,594 articles were independently screened in two stages by two reviewers (A.H. and N.M.), with a third acting as a tiebreaker within the screening software Covidence. First, titles and abstracts were screened against preset inclusion criteria after discussion and consensus among all authors and approved by the principal investigator (M.M). The full text of 18 eligible or potentially eligible studies was screened against the inclusion criteria, and 10 articles were included that reported data on the safety and efficacy of T-DXd in GI malignancies (**Figure 2**), excluding hepatocellular carcinoma (HCC). The inclusion criteria were:

1. Original studies (clinical trials and case-control, retrospective, and prospective cohort studies);
2. Articles reporting data for adult patients aged 18 years and above;
3. Studies reporting the safety and efficacy of T-DXd only in HER2-positive [HER2 + 3 by immunohistochemistry (IHC) or HER2 + 2 by IHC/ISH+] GI malignancies.

Studies with HCC as a diagnosis were excluded, given there was no expression of HER2. Studies with HER2–0 or +1 by IHC were also excluded. Eight studies were excluded in the secondary review, all of which were found to be duplicates or irrelevant and did not report data on T-DXd AEs or outcomes (**Figure 1**). **Supplementary Table S1** lists excluded studies along with the reasons for exclusion.

### Data extraction

Five authors (M.P., A.T., S.V., A.A., and A.D.) independently extracted data from the 10 selected studies. A.H. and S.I. verified the data for any discrepancies. Data were collected on baseline characteristics (i.e., number of patients, sex, age, diagnosis, HER2-expression status, T-DXd dosing, and prior lines of therapies); efficacy, i.e., ORR, complete response (CR), partial response (PR), median follow-up, OS, PFS, and treatment-related mortality (TRM); and AEs, i.e., anemia, neutropenia, febrile neutropenia, thrombocytopenia, nausea/vomiting, diarrhea, elevation in liver function test, pneumonitis/ILD, heart failure, and malaise/fatigue. The baseline characteristics of each study are listed and tabulated in **Table 1**.

**Table 1:** Baseline characteristics (n=653).

	Cutsem et al., 2023 (16)	Shen et al., 2023 (17)	Shitara et al., 2019 (18)	Yoshino et al., 2022 (19)	Ohba et al., 2022 (20)	Mastumoto et al., 2022 (21)	Raghav et al., 2023 (22)	Yamaguchi et al., 2022 (23)	Shitara et al., 2024 (24)	Shitara et al., 2020 (4)
**Patients, n**	79	73	44	86	22	18	122	20	64	125
**Male, n (%)**	57 (72)	NA	32 (73)	46 (53.5)	NA	13 (72)	64 (52)	16 (80)	50 (78)	70 (76)
**Median age, years (range)**	60.7(52–68)	48(65.8)*	68(62.5–72)	58.5(27–79)	NA	71(51–85)	NA	64 (29–74)	65.5 (37–84)	65 (34–82)
**Type of study**	Phase II	Phase II	Phase I	Phase II	Phase II	Retrospective	Phase II	Phase II	OLEA	Phase II
**Region**	USA, Europe	China	USA, Japan	Asia, Europe, North America	Japan	Japan	USA, Japan, Belgium, Italy	Japan, South Korea	Japan	Japan, South Korea
**Location of malignancy**	Gastric & GEJ	Gastric & GEJ	Gastric & GEJ	Colorectal	GB & Biliary	Gastric	Colorectal	Gastric & GEJ	Gastric & GEJ	Gastric & GEJ
**HER2 + 3 IHC**	68 (86)	52 (72)	36 (82)	40 (46.5)	10 (45.5)	14 (78)	99 (81.5)	0	47 (73.4)	96 (77)
**HER2 + 2/FISH+**	10 (13)	21 (28)	8 (18)	28 (32.6)	12 (54.5)	4 (22)	23 (18.5)	20 (100)	17 (26.6)	29 (23)
T-DXd dose, n (%)										
*5.4 mg/kg*	0	0	19 (43)	0	22 (100)	0	82 (67)	0	27(42)**	0
*6.4 mg/kg*	79 (100)	73 (100)	25 (57)	86 (100)	0	18 (100)	40 (33)	20 (100)	64 (100)***	125 (100)
**Prior no. of therapies, median (range)**	2 (1–3)	2 (2–6)	3 (2–5)	4 (NA)	2 (1–4)	3 (NA)	4 (NA)	2 (2–4)	4 (2–11)	2 (NA)
**Prior HER2 Rx, n (%)**	79 (100)	NA	44 (100)	16 (18.6)	NA	NA	11 (9)	NA	64 (100)	125 (100)

n, number; NA, not available; HER-, human epidermal growth factor receptor negative; IHC, immunohistochemistry; FISH, fluorescence *in situ* hybridization; Rx, treatment; GEJ, gastroesophageal function, OLEA, open-label expanded access; GB, hall bladder.

*<65 years.

**After 1st cycle.

***Only with 1st cycle.

### Quality evaluation

The methodological quality of the included studies was evaluated using the National Institute of Health (NIH) quality assessment tool and all the studies were rated as good. **Supplementary Table S2** lists the quality and risk of bias assessment.

### Statistical analysis

A pooled analysis was performed using the ‘meta’ package (Schwarzer et al., R programming language), and proportions with 95% confidence intervals (CIs) were computed. The inter-study variance was calculated using the Der Simonian–Laird Estimator.

## Results

We identified a total of 10 studies with 653 patients using T-DXd for the treatment of GI malignancies. The median age of patients was 64.5 years (27–85) (4, 16–19, 21, 23, 24), and 53% were male (4, 16, 18, 19, 21–24). Furthermore, 70% of these 653 patients were HER2 + 3 by IHC, and 26% of patients were HER2 + 2 by IHC/FISH. A 5.4 mg/kg T-DXd dose was given to 59.5% (150/252) of the patients (18, 20, 22, 24). Patients in one study received the 5.4mg/kg dose after the first cycle (24). Moreover, a 6.4 mg/kg T-DXd dose was used in 84% (530/631) of the patients (4, 16–19, 21–24). The patients in all studies received at least 2 (1–11) prior therapies and 65% (339/520) of the patients had prior HER2 treatment (4, 16, 18, 19, 22, 24).

### Outcomes

The primary endpoints were objective response rate (ORR) and safety in Cutsem et al. (2023) and Shitara et al. (2019) (16, 18). The sole primary endpoint was ORR in seven studies (4, 17, 19–23). Shitara et al. (2024) reported safety as the primary endpoint (24). The median follow-up was 5.9 months (0.5–30.5) (16–19, 21–23). Median OS was 11.15 months (1.4–20.8) (4, 16–21, 23). PFS was 5.6 months (2.6–8.7) (4, 16–23). The median duration of response (DoR) was 7 (0.7–22.3) months (4, 16, 18, 19, 22).

The overall pooled ORR was 36.9% (95% CI 0.315–0.425, I2 = 41%, n = 589) (4, 16–23). The pooled ORR for a 5.4 mg/kg dose of T-DXd was 23.4% (95% CI 0.135–0.350, I2 = 57%, n = 188). The pooled ORR for a 6.4 mg/kg dose of T-DXd was 31.3% (95% CI 0.202–0.435, I2 = 88%, n = 567). The CR and PR rates were 1.3% (95% CI 0.000–0.047, I2 73%, n = 516) and 35.2% (95% CI 0.311–0.395, I2 = 0%, n = 516), respectively (4, 16, 18–23). The pooled rates of stable disease and progressive disease were 34.4% (95% CI 0.276–0.415, I2 = 42%, n = 372) and 11.6% (95% CI 0.075–0.165, I2 = 35%, n = 372), respectively (4, 16, 18, 19, 21, 23). Treatment-related mortality was 2.9% (95% CI 0.004–0.069, I2 = 72%, n = 491) (4, 16–19, 23, 24). The outcomes of each study are listed in **Table 2**.

**Table 2:** T-DXd outcomes (n=653).

	Cutsem et al., 2023 (16)	Shen et al., 2023 (17)	Shitara et al., 2019 (18)	Yoshino et al., 2022 (19)	Ohba et al., 2022 (20)	Mastumoto et al., 2022 (21)	Raghav et al., 2023 (22)	Yamaguchi et al., 2022 (23)	Shitara et al., 2024 (24)	Shitara et al., 2020 (4)
**Primary endpoint**	ORR, Safety	ORR	ORR, Safety	ORR	ORR	ORR	ORR	ORR	Safety	ORR
**Secondary endpoint**	PFS, OS, Death	PFS, OS, DoR	PFS, OS	PFS, OS, DoR, Safety	PFS, OS, Safety	PFS, OS	PFS, OS, DoR	PFS, OS	NA	PFS, OS, DoR, Safety
**ORR, n (%)**	30 (38)	21 (28.8)	19 (43.2)	24 (45.3)	8 (36.4)	7 (41)	42 (21)	8 (42)	NA	61 (51)
**CR, n (%)**	3 (4)	NA	0	0	2 (9)	0	0	0	NA	11 (9)
**PR, n (%)**	27 (34)	NA	19 (43)	24.45.3)	6 (27)	7 (41)	42 (21)	8 (42)	NA	50 (42)
**OS, months (range)**	12.1(8.6–NE)	10.2 (7.5–14.3)	12.8 (1.4–25.4)	15.5 (8.8–20.8)	7.1 (4.7–10.6)	6.5 (3.7–10.6)	NA	7.8 (4.7–NE)	NA	12.5 (9.6–14.3)
**PSF, months (range)**	5.5 (4.2–7.2)	5.7 (4–6.8)	5·6 (3–8·3	6.9 (4.1–8.7)	4.4 (2.8–8.3)	3.9 (2.6–7.7)	5.8 (4.2–7)	4.4 (2.7–7.1)	NA	5.6 (4.3–6.9)
**Median DoR, months (range)**	8.1 (4.1–NE)	NA	7 (4.4–16.6)	7 (5.8–9.5)	NA	NA	5.5 (3.7–8.1)	NA	NA	4.6 (0.7–22.3)
**SD, n (%)**	34 (43)	NA	16 (36)	20 (37.7)	NA	6 (33)	NA	9 (47)	NA	42 (35)
**PD, n (%)**	13 (16)	NA	9 (20)	5 (9.4)	NA	2 (11)	NA	2 (10)	NA	14 (12)
**TRM, n (%)**	2 (3)	0	2 (4.5)	9 (10.5)	NA	NA	NA	0	6 (9.4)	1 (<1)
**Median F/U (range) months**	5.9 (4.6–8.6)	8 (6–13.2)	5.5(2.8–13.1)	14.4 (1.2–26.8)	NA	5.8(0.5–30.5)	9.6 (0.5–17.1)	2.8(0.7–14.9)	NA	NA

T-DXd, trastuzumab feruxtecan; PFS, progression-free survival; OS, overall survival; DoR, duration of response; NA, not available; ORR, objective response rate; CR, complete response; PR, partial response; SD, stable disease; PD, progressive disease; TRM, treatment-related mortality; F/U, follow-up.

### Safety analysis

#### Dose-based adverse events

AEs, including anemia, neutropenia, febrile neutropenia, thrombocytopenia, diarrhea, nausea, ILD/pneumonitis, heart failure, hepatitis, and malaise/fatigue, were reported in the studies. The treatment was discontinued because of toxicities in 77 (15%) patients (4, 16–20, 23, 24). AEs of any grade for a 5.4 mg/kg dose were not specified by any study. A total of 272 AEs of any grade were reported for a 6.4 mg/kg dose. The number of grade 3/4 SAEs for a 5.4 mg/kg dose was 67. There were 146 total grade 3/4 SAEs for a 6.4 mg/kg dose.

#### Pooled rate of adverse events

The pooled rate for any grade of anemia was 36.6% (95% CI 0.220–0.525, I2 = 93%, n = 580) (4, 16, 18–24), while for grade 3/4 anemia it was 17.4% (95% CI 0.075–0.300, I2 = 90%, n = 494) (4, 16, 18, 19, 21–24). The pooled rates of any grade and grade 3/4 neutropenia were 32.5% (95% CI 0.177–0.493, I2 = 93%, n = 516) and 18.1% (95% CI 0.051–0.363, I2 = 95%, n = 494) (4, 16, 18–23), respectively. The pooled rates of any grade and grade 3/4 thrombocytopenia were 18.5% (95% CI 0.085–0.312, I2 = 91%, n = 558) and 3.3% (95% CI 0.004–0.081, I2 = 80%, n = 558) (4, 16, 18, 19, 21–24), respectively. The pooled rate of any grade diarrhea was 22.7% (95% CI 0.147–0.318, I2 = 81%, n = 558) (4, 16, 18, 19, 21–24), and for grade 3/4 diarrhea it was 0.6% (95% CI 0.000–0.016, I2 = 0%, n = 558) (4, 16, 18, 19, 21–23). The pooled rates of any grade and grade 3/4 nausea were 47.9% (95% CI 0.294–0.667, I2 = 95%, n = 558) and 3.7% (95% CI 0.018–0.060, I2 = 23%, n = 558) (4, 16, 18, 19, 21–24), respectively. The pooled incidences of any grade and grade 3/4 pneumonitis/ILD were 8.8% (95% CI 0.057–0.124, I2 = 49%, n = 635) and 1.6% (95% CI 0.002–0.040, I2 = 58%, n = 635) (4, 16–20, 22–24), respectively. The pooled rate of any grade of heart failure was 2.3% (95% CI 0.000–0.084, I2 = 74%, n = 286) (4, 16, 18, 21, 23). There were no grade 3/4 heart failure events (4, 16, 18, 21, 23). The pooled incidences of any grade and grade 3/4 hepatitis were 1.8% (95% CI 0.002–0.045, I2 = 56%, n = 434) and 0.2% (95% CI 0.000–0.011, I2 = 0%, n = 434) (4, 16, 18, 22, 24), respectively. The total number of events for each study is mentioned in **Table 3**.

**Table 3:** T-DXd adverse events (n=653).

	Cutsem et al., 2023 (16)	Shen et al., 2023 (17)	Shitara et al., 2019 (18)	Yoshino et al., 2022 (19)	Ohba et al., 2022 (20)	Mastumoto et al., 2022 (21)	Raghav et al., 2023 (22)	Yamaguchi et al., 2022 (23)	Shitara et al., 2024 (24)	Shitara et al., 2020 (4)
Anemia, n (%)										
*All grades*	19 (24)	NA	18 (41)	31 (36)	17 (53)	6 (33)	38 (31)	10 (50)	1 (1.6)	72 (58)
*Grade 3/4*	11 (14)	NA	13 (30)	12 (14)	NA	2 (11)	3 (2)	6 (30)	0	42 (38)
Neutropenia, n (%)										
*All grades*	4 (5)	NA	12 (27)	26 (30)	10 (31)	5 (28)	35 (28)	9 (45)	NA	79 (63)
*Grade 3/4*	4 (5)	NA	9 (20)	19 (22)	0	3 (17)	3 (2)	5 (25)	NA	64 (51)
NF, n (%)										
*All grades*	0	NA	11 (25)	NA	NA	NA	8 (6)	3 (15)	6 (9.6)	6 (5)
*Grade 3/4*	2 (2.5)	NA	NA	NA	NA	NA	3 (2)	0	NA	6 (5)
Decreased platelets, n (%)										
*All grades*	4 (5)	NA	15 (34)	28 (32.6)	NA	2 (11)	29 (24)	3 (15)	1 (1.6)	49 (39)
*Grade 3/4*	1 (1)	NA	8 (18)	8 (9.3)	NA	0	1 (1)	0	0	12 (10)
Diarrhea, n (%)										
*All grades*	28 (35)	NA	9 (20)	23 (26.7)	NA	3 (20)	30 (25)	6 (30)	2 (3.1)	40 (32)
*Grade 3/4*	1 (1)	NA	0	1 (1.2)	NA	0	1 (1)	0	NA	3 (2)
Nausea/vomiting, n (%)										
*All grades*	47 (59)	NA	31 (70)	53 (62)	NA	5 (28)	70 (57)	11 (55)	1 (1.6)	79 (63)
*Grade 3/4*	6 (8)	NA	1 (2)	5 (6)	NA	1 (6)	5 (4)	1 (5)	0	6 (5)
ILD/pneumonitis, n (%)										
*All grades*	9 (10)	3 (3.2)	3 (7)	8 (9)	8 (25)	NA	12 (10)	1 (5)	3 (4.7)	12 (10)
*Grade 3/4*	2 (2.5)	0	1 (2)	4 (4)	4(12.5)	NA	0	0	1 (1.6)	3 (2)
Decrease in EF, n (%)										
*All grades*	6 (11)	NA	1 (2)	NA	NA	2 (11)	NA	0	NA	0
*Grade 3/4*	0	NA	0	NA	NA	0	NA	0	NA	0
Hepatitis/LFT elevation, n (%)										
*All grades*	2 (2)	NA	3 (7)	NA	NA	NA	3 (2)	NA	1 (1.6)	0
*Grade 3/4*	1 (1)	NA	0	NA	NA	NA	1 (1)	NA	0	0
Malaise/fatigue n (%)										
*All grades*	30 (38)	NA	15 (34)	31 (36)	NA	11 (61)	31 (25)	9 (45)	NA	27 (22)
*Grade 3/4*	3 (4)	NA	0	1 (1.2)	NA	0	3 (2)	2 (10)	NA	9 (7)
**Drug stopped because of toxicities, n (%)** **/toxicity type**	8 (10)/ILD	17 (23)/*	6 (14)/NA	13 (15)/ILD	8 (25)/NA	NA	NA	2 (10)/1 ILD, 1 other	5 (7.8)/NA	18 (15)/NA

T**-**DXd, trastuzumab deruxtecan; NA, not available; n, number of patients; NF, neutropenic fever; ILD, interstitial lung disease; LFT, liver function test.

*COIVD-19 and other adverse events.

## Discussion

HER2, a proto-oncogene expressed on many solid tumors, including those in the breast, kidney, and GI tract, is a therapeutic target (25–27). Apart from breast cancer, there is significant variability in HER2 expression and response to anti-HER2 therapy. Trastuzumab and chemotherapy have demonstrated some effectiveness in GI malignancies, especially esophagogastric cancers. ADCs enable more precise delivery of a high cytotoxic payload combined via a stable linker with specific monoclonal antibodies. T-DXd has shown effectiveness across many different cancer types including HER2-low and -ultra-low breast cancers (28). In our single-arm meta-analysis, T-DXd demonstrated a pooled ORR of 36.9% (31–42) as a second or higher line of treatment for HER2-positive GI malignancies. This evidence underscores the potential of T-DXd to enhance clinical outcomes for this challenging patient population. The highest ORR for gastric and gastroesophageal junction tumors was 51%, according to Shitara et al. (2020) (4). In colon cancer, the best ORR was 45%, as reported by Yoshino et al. (19). In contrast, the ORR for T-DXd in breast and lung cancer has previously been reported at 66.9% and 55.6%, respectively (29). This indicates a lower ORR of T-DXd in GI malignancies when compared to HER2+ breast and lung cancer. The disease control rate (DCR) for gastric tumors ranged from 76% to 89.5%, as noted by Matsumoto and Yamaguchi et al. (21, 23). Conversely, the DCR for breast cancer has been reported at 96.5% (29), suggesting that HER2 GI malignancies have a slightly lower DCR in comparison to breast cancer.

The average age of patients with gastric cancer is 68 years, while colon cancer is typically diagnosed at 70 years (30, 31). Matsumoto et al. reported outcomes in the oldest population with a median age of 71 (51–85) in our included studies (21). Their results showed that age alone did not significantly affect cancer outcomes. Similarly, Yoshino et al. found no significant difference in ORR between patients older than 65 (42.9%) and those younger than 65 (50.0%) (19). This data confirms that age does not appear to affect treatment response adversely. Gastric cancers are more common in men, with a male-to-female ratio ranging from 2:1 to 3:1 (32). In our analysis, the participants diagnosed with gastric cancer were predominantly male, accounting for 72% to 80% of the cases.

Poor functional status, as indicated by an Eastern Cooperative Oncology Group performance status (ECOG PS) greater than 2, is a well-known adverse prognostic factor for GI malignancies (33, 34). In our analysis, Yoshino et al. found that the ORR was significantly better for patients with an ECOG score of 0 at 54% (range 37%–70%) compared to those with an ECOG score of 1, which had an ORR of only 25% (range 7.3%–52.4%) (19). Additionally, Yamaguchi et al. reported that patients with HER2 IHC 1+ experienced more deterioration in ECOG scores over a 12-month period than those with HER2 IHC 2+/ISH- (23). Only Matsumoto et al. focused on patients with ECOG scores of 2 or 3; however, their study had a very small sample size (n=3), leaving recommendations for the use of T-DXd in patients with poorer health and lower performance status unclear (21).

Three studies [Yoshino et al. (19), Yamaguchi et al. (23), Shitara et al., (4)] evaluated the ORR in relation to HER2 status in tumors. Yoshino et al. found that metastatic colon cancer patients with HER2 IHC 3+ or HER2 2+/ISH positive status exhibited a PR rate of 45%. In contrast, no PR was observed in patients with HER2 2+/ISH negative status. The DoR was significantly longer in the HER2 IHC 3+ or HER2 2+/ISH-positive group, averaging 7 months (ranging from 5.8 to 9.5 months), while the HER2 2+/ISH-negative group showed no response (19). Similarly, Shitara et al. (2020) reported an improved ORR of 58% in patients with HER2 IHC 3+ disease (n=47), compared to a 29% response rate in those with HER2 IHC 2+ and ISH-positive disease (4). Conversely, Yamaguchi et al. noted a PR of 26% in HER2 2+/ISH negative gastric cancer patients, indicating that clinical responses to trastuzumab may vary among different GI malignancies (23). Three studies [Yoshino et al. (19), Yamaguchi et al. (23), Shitara et al., (4)] showed no CR in HER2 + GI malignancies (4, 19, 23). Only Shitara et al. (2020) reported that 9% (n=11) of the participants achieved a CR compared to physician-choice chemotherapy (4). Similarly, Cutsem et al. showed that 5% (n=4) of the patients had a CR (16).

Yoshino et al. reported a better median OS of 15.5 months (8.8 to 20.8 months) for patients in the HER2 3+ IHC or HER2 2+/ISH+ group (n=53). In contrast, patients in the HER2 2+/ISH- group (n=15) had a median OS of only 7.3 months (range: 3.0 to not evaluable). Additionally, Yoshino et al. found that PFS was better for those with HER2 IHC 3+ or HER2 2+/ISH+ at 8.3 months compared to 4.1 months for the HER2 2+/ISH- group (19).

In our analysis, 9 studies (except Ohba et al.) assessed the efficacy and safety of a 6.4 mg/kg dose, while four studies [Shitara et al., (18), Ohba et al., (20), Raghav et al., (22)] assessed a 5.4 mg/kg dose, suggestive greater consensus on utilizing 6.4 mg/kg dose. Raghav et al. reported different ORRs depending on the dose of T-DXd. The author(s) showed a better PR of 38% (27%–49%) among the 5.4 mg/kg arm and 27.5% (14.6%–43.9%) among the 6.4 mg/kg arm. The study also showed worse PFS with a high dose (6.4 mg/kg) at 5.5 months (4.2–7.0) vs. 5.8 months (4.6–7.0) with a lower dose (5.4 mg/kg) (22). Contrary to that, Shitara et al. (2019) showed better ORR with a 6.4 mg/kg dose at 52% (31%–72%) and only 31% (6%–56%) with a 5.4 mg/kg dose (18).

Interestingly, Matsumoto et al. calculated outcomes of HER2+ advanced gastric cancer and prior treatment with immune checkpoint inhibitors (ICIs). The author (s) showed better median PFS/OS among patients with gastric cancer treated with T-DXd and prior ICI treatment. The PFS with ICI exposure (n=11) was 6.5 months and only 2.9 months with no ICI exposure (n=7), p=0.02. OS was 9.2 months with ICI (n=11) and 3.7 months without ICI (n=7), p=0.08. Similarly, ORR was 60% vs. 14%, p=0.059, among patients with prior ICI exposure (21). This finding suggests that a concomitant or earlier treatment with ICIs among HER2+ GI malignancies could potentiate the beneficial effects of T-DXd. Matsumoto et al. showed a DCR of 76%; this DCR was higher in patients with prior ICI treatment at 90% vs. 57% with no prior ICIs, p=0.11 (21).

Left-sided colon cancer has a slightly better 5-year survival at 68.4% compared to 65.6% in right-sided colon cancer (35, 36). In our analysis, PFS and ORR depending on the location of colon cancer were reported by Yoshino et al. The author(s) showed that a left-sided HER2+ colon tumor had better PFS (7.3 months vs. 3.5 months) compared to a right-sided tumor (cecum and ascending and transverse colon) along with better ORR at 46.8% compared to 33% (4.3%–77.7%), respectively (19).

The presence of ascites in GI malignancies is regarded as a poor prognostic factor (37). In our included studies, the role of ascites was not evaluated due to the nature of phase 1 and 2 studies, except in the case of Matsumoto et al. The authors reported that 44% (n=8) of the patients had ascites, with 7 out of 8 experiencing massive ascites related to HER2+ advanced gastric cancer. The PFS was shorter for these patients at 2.75 months compared to 6.5 months for those without ascites (p=0.01). Similarly, the OS was also significantly worse, averaging 3.9 months for patients with ascites vs. 9.4 months for those without (p=0.04) (21).

In our analysis, most participants discontinued T-DXd treatment due to disease progression. According to Cutsem et al., approximately 75% of patients stopped T-DXd because their disease worsened (16). Shitara et al. (2020) found that 15% of patients discontinued therapy due to side effects (4). Additionally, Shitara et al. (2019) and Yoshino et al. reported 75% and 70% treatment discontinuation due to disease progression, respectively (18, 19).

We also analyzed the safety profile of T-DXd among HER2+ GI malignancies. Our analysis showed better tolerability of T-DXd treatment among HER2+ GI malignancy participants. The most reported side effect of T-DXd treatment was nausea, with a pooled incidence of 47.9%. Shitara et al. (2020) reported nausea among 63% (n=79) of the participants. Fortunately, grade 3/4 nausea was only reported by 5% (n=6) (4). Dowling et al. showed that the prevalence of all-grade nausea caused by T-DXd in HER2+ breast cancer patients was 76% (38). ILD/pneumonitis is a less common but serious side effect of monoclonal antibody therapy against the HER2 receptor. Trastuzumab causes ILD in approximately 9.9% of patients. Similarly, T-DM1 causes ILD in only 1.1% of cases. Lapatinib showed an ILD incidence of 0.2% (39). Raghav et al. showed drug-related ILD to be more frequent with a 6.4 mg/kg dose (12.8%) compared to 8.4% with a 5.4 mg/kg dose. The incidence of ILD seems to correlate with higher doses of T-DXd (22). Matsumoto et al. showed drug-induced grade 2 heart failure in 6% of the patients (21). Shitara et al., 2019 reported one case of decreased EF (grade 2) and one with prolonged QT interval (grade 3) (18). Overall, the included patients had no significant cardiac side effects.

Our single-arm meta-analysis has several limitations. The diverse range of GI malignancies among the included studies makes it challenging to generalize our pooled data. Additionally, most of the studies were phase 1 and phase 2 trials with relatively small sample sizes. The absence of significant randomization and potential selection bias may compromise the quality of the data in our analysis.

This systemic review shows that T-DXd is an effective and relatively safe therapeutic option for HER2-positive advanced GI malignancies. T-DXd demonstrates a moderate ORR and a clinically meaningful benefit even in patients who have experienced disease progression after multiple lines of therapies for GI malignancies. The safety data shows no new signals. ILD is a rare but serious side effect that can be managed effectively with early diagnosis and prompt intervention. Further studies are required to leverage the potential of ADCs, which demonstrate effectiveness in HER 2-low and -ultra-low GI cancers, ADC combinations, and optimal sequencing of these agents in advanced GI cancers.

## Data Availability

The original contributions presented in the study are included in the article/**Supplementary Material**. Further inquiries can be directed to the corresponding author.
