# Supplementary material for: Efficacy and safety of trastuzumab deruxtecan in gastrointestinal malignancies: a systemic review and meta-analysis
Source: Front Gastroenterol (Lausanne). 2025 Apr 29;4:1559934. doi: 10.3389/fgstr.2025.1559934 (PMC12952437; doi:10.3389/fgstr.2025.1559934)
Supplement: Supplementary file 1 [file Table1.docx]

***Table S1***

***Excluded studies with reason***

| **Sr. No** | **Studies** | **Reason** |
| --- | --- | --- |
| 1 | A phase II, multicenter, open-label study of trastuzumab deruxtecan (T-DXd; DS-8201) in patients (pts) with HER2-expressing metastatic colorectal cancer (mCRC): DESTINY-CRC01  DOI: [10.1200/JCO.2020.38.15_suppl.4000](https://dx.doi.org/10.1200/JCO.2020.38.15_suppl.4000) | Duplicate |
| 2 | P-139 Trastuzumab deruxtecan showed similar clinical benefit for frail HER2 positive gastric cancer patients: Retrospective observational study  DOI: [10.1016/j.annonc.2022.04.229](https://dx.doi.org/10.1016/j.annonc.2022.04.229) | Retrospective study very limited data, no information for methods or toxicity |
| 3 | Multicenter phase II study of trastuzumab deruxtecan (DS-8201) for HER2-positive unresectable or recurrent biliary tract cancer: HERB trial  DOI: [10.1200/JCO.2020.38.15_suppl.TPS4654](https://dx.doi.org/10.1200/JCO.2020.38.15_suppl.TPS4654) | Only Study design, no data mentioned |
| 4 | 1205MO Updated analysis of DESTINY-Gastric02: A phase II single-arm trial of trastuzumab deruxtecan (T-DXd) in western patients (Pts) with HER2-positive (HER2+) unresectable/metastatic gastric/gastroesophageal junction (GEJ) cancer who progressed on or af  DOI: [10.1016/j.annonc.2022.07.1323](https://dx.doi.org/10.1016/j.annonc.2022.07.1323) | Duplicate only updates provided, will copy updated results only |
| 5 | Trastuzumab Deruxtecan (T-DXd) in Patients (Pts) With HER2-Positive Gastric Cancer (GC) or Gastroesophageal Junction (GEJ) Adenocarcinoma Who Have Progressed On or After a Trastuzumab-Containing Regimen (DESTINY-Gastric04, DG-04): A Randomized Phase 3 Stu  DOI: [10.1159/000521004](https://dx.doi.org/10.1159/000521004) | Only Study design, no data mentioned |
| 6 | A phase II trial of [fam-] trastuzumab deruxtecan (T-DXd, DS-8201a) in subjects with HER2- positive, unresectable, or metastatic gastric or gastroesophageal junction (GEJ) adenocarcinoma  DOI: [10.1200/JCO.2020.38.4_suppl.TPS460](https://dx.doi.org/10.1200/JCO.2020.38.4_suppl.TPS460) | Only Study design, no data mentioned |
| 7 | Dose-escalation and dose-expansion study of trastuzumab deruxtecan (T-DXd) monotherapy and combinations in patients (pts) with advanced/metastatic HER2+ gastric cancer (GC)/gastroesophageal junction adenocarcinoma (GEJA): DESTINY-Gastric03  DOI: [10.1159/000521004](https://dx.doi.org/10.1159/000521004) |  |
| 8 | Trastuzumab deruxtecan (DS-8201) in patients with HER2-expressing metastatic colorectal cancer (DESTINY-CRC01): a multicentre, open-label, phase 2 trial.  DOI: [10.1016/S1470-2045(21)00086-3](https://dx.doi.org/10.1016/S1470-2045(21)00086-3) **·** Ref ID: 33961795 | Duplicate |
